# Potential of Zinc Oxide Nanostructures in Biosensor Application

**DOI:** 10.3390/bios15010061

**Published:** 2025-01-18

**Authors:** Ibrahim M. Maafa

**Affiliations:** Department of Chemical Engineering, College of Engineering and Computer Sciences, Jazan University, Jazan 45142, Saudi Arabia; imoaafa@jazanu.edu.sa

**Keywords:** ZnO nanostructures, biosensors, medical diagnostics

## Abstract

The burgeoning field of biosensors has seen significant advancements with the induction of zinc oxide (ZnO) nanostructures, because of their unique structural, electrical, and optical properties. ZnO nanostructures provide numerous benefits for biosensor applications. Their superior electron mobility enables effective electron transfer between the bioreceptor and transducer, enhancing sensitivity and reducing detection limits. Furthermore, ZnO’s biocompatibility and non-toxicity make it ideal for in vivo applications, reducing the chances of adverse biological responses. This review paper explores the prospects of ZnO nanostructures in the development of biosensors, focusing on their morphological and structural characteristics. Various synthesis techniques, that include sol-gel, sputtering, and chemical vapor deposition, were successfully employed to prepare different ZnO nanostructures, like nanorods, nanotubes, and nanowires. The various findings in this field underscore the efficacy of ZnO nanostructures in enhancing the specificity and sensitivity of biosensors, presenting a promising avenue for the advancement of point-of-care diagnostic devices.

## 1. Introduction

**Figure 1:**
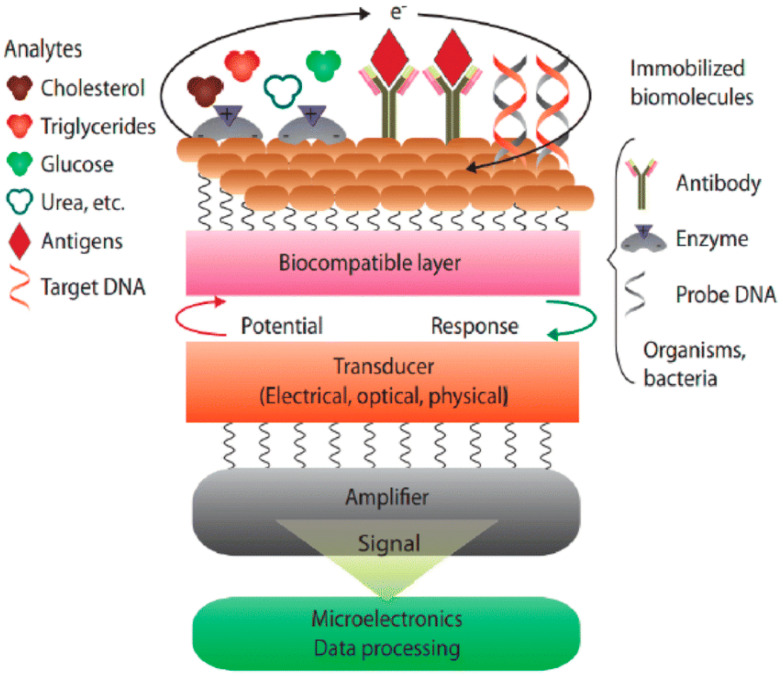
Sketch showing the various components of biosensor. Adapted from [1].

Biosensors are devices that analyse and transform a biological reaction into an electrical signal. They are employed to identify the presence or concentration of biological analytes such as antibodies, microorganisms, enzymes, or nucleic acids. Biosensors combine a biological component with a physicochemical detector, allowing them to provide qualitative and quantitative analytical information. A sketch of a biosensor detailing its functioning is displayed in Figure 1. Recently, ZnO nanostructures have garnered significant attention due to their remarkable chemical and physical properties, which make them ideal candidates for a broad spectrum of applications, including biosensors. The capability to synthesize ZnO in various nanostructured forms, such as nanowires, nanorods, nanotubes, nanoflowers, and nanoparticles, further enhances its versatility and functionality in biosensor applications. The incorporation of ZnO nanostructures into biosensors has been driven by the need for highly sensitive, stable, and cost-effective detection methods for medical diagnostics, food safety, and environmental surveillance. The high surface area-to-volume ratio of ZnO nanostructures provides an extensive active surface for the anchoring of biomolecules, leading to enhanced interaction with target analytes and improved sensor performance.

ZnO nanostructures offer several advantages in biosensor applications. Their exceptional electron mobility facilitates efficient electron transfer between the bioreceptor and the transducer, leading to lower detection limits and increased sensitivity. Additionally, ZnO is biocompatible and non-toxic, making it suitable for in vivo applications and minimizing adverse biological reactions. The material’s thermal and chemical stability ensure reliable operation under various environmental conditions, while its cost-effectiveness makes it an attractive option for widespread use. Despite these advantages, several challenges must be addressed to fully exploit the capability of ZnO nanostructures in biosensors. Achieving consistent and reproducible fabrication of ZnO nanomaterials. with desired properties remains a critical issue. Furthermore, effective surface functionalization techniques are necessary to immobilize bioreceptors on ZnO nanomaterials without compromising their activity or stability. Additionally, the enduring stability of ZnO nanomaterials in biological environments must be ensured to maintain sensor performance over extended periods.

The present article furnishes a thorough overview of the current scenario of research on ZnO nanostructures for biosensor applications. We begin by discussing the importance of biosensors in conducting medical diagnostics, highlighting their advantages. We then explore the various detection strategies employed in the working of biosensors and the integration of ZnO nanostructures in its development. Finally, we address the use of ZnO nanostructures in various kinds of biosensors. By synthesizing the latest advancements, this review endeavours to provide a valuable source for researchers and practitioners in the area, fostering the advancement of next-generation biosensors that leverage the unique characteristics of ZnO nanostructures for enhanced detection capabilities.

## 2. Importance of Biosensors in Diagnostics

Biosensors have become integral to modern diagnostics, offering significant advantages over traditional methods. Their importance in diagnostics is fueled by their capability to provide rapid, accurate, and cost-effective detection of various analytes, making them essential tools in medical, environmental, and food safety applications. Biosensors provide near-real-time or real-time results, which is important for timely medical decision-making, particularly in situations such as infectious disease outbreaks or emergency medical conditions where time is of the essence. Additionally, biosensors enable point-of-care testing, allowing diagnostics to be performed at the patient’s bedside, in remote locations, or even at home, thus reducing the requirement for complex laboratory infrastructure and speeding up the diagnostic process.

Biosensors are engineered to identify very low concentrations of analytes, making them highly sensitive, which is important for the early identification of diseases and significantly improving treatment outcomes. The use of specific bioreceptors, such as antibodies, enzymes, or nucleic acids, ensures that biosensors can accurately identify target analytes amidst a complex mixture of substances, reducing the likelihood of false positives or negatives [2,3]. Furthermore, biosensors can be tailored to detect a variety of biological molecules, including glucose, cholesterol, pathogens, toxins, and genetic markers, making them useful in numerous diagnostic applications, from monitoring chronic diseases to detecting acute infections. Some biosensors can simultaneously identify several analytes in one test, providing comprehensive diagnostic information from a single sample.

The miniaturization and automation of biosensor technology have significantly reduced the costs associated with diagnostic testing, making diagnostics more accessible, especially in settings with limited resources. Biosensors typically require smaller amounts of reagents in contrast to conventional laboratories methods, further reducing operational costs. Many biosensors are designed to be user-friendly, requiring minimal training to operate, which makes them befit for use by non-specialists, such as patients or primary care providers. The compact and portable nature of many biosensors enhances their usability in various settings, including fieldwork and home healthcare.

Biosensors often use minimally invasive or non-invasive sampling methods, for instance saliva, urine, or sweat, which are more comfortable for patients compared to traditional blood draws or biopsies. This ease of non-invasive testing allows for more frequent monitoring of patients, leading to better disease management and more personalized healthcare. Modern biosensors can be incorporated in digital health platforms, enabling the uninterrupted surveillance of health parameters and the transmission of data to healthcare providers. This connectivity facilitates remote patient monitoring and telemedicine applications. The data generated by biosensors can be analyzed using big data analytics and artificial intelligence (AI) to identify trends, predict disease outbreaks, and personalize treatment plans.

The glucose sensor is perhaps the most renowned example of a biosensor currently in use for managing diabetes; another widely used biosensor is the home pregnancy test [4,5]. Biosensors offer the capability of an easy-to-use and cost-effective technological framework for identification of infectious diseases, allowing for the timely identification of pathogens and appropriate treatment [6]. Advances in nanotechnology have resulted in the advancement of biosensors capable of performing complex diagnostic assays. The evolution of biosensors in the medical diagnosis represents the most favourable approaches to address the increased demand of highly sensitive, rapid, and economical analytical methods.

## 3. Detection Strategy of Biosensors

Biosensors are categorized into two distinct groups based on the detection strategy i.e., label-based and label-free detection [7]. Label-based biosensors are regarded as more reliable, but they require joint action of specific sensing element coupled with the target protein that are immobilized. In contrast, label-free methods enable the detection of target molecules without the need for labeling or tagging, even when it is challenging. Recent advancements in biotechnology, driven by multidisciplinary efforts, have paved the way for the development of label-free biosensors with numerous applications in medicine and environmental science [8]. Most common bottleneck encountered in the advancement of biosensor is the insufficient reproducibility and stability of their interfaces (physico-chemical properties) [9]. Among the electrochemical biosensors, glucometer is first in the line of discovery [10]. At present electrochemical biosensors are leading the field, primarily focusing on the monitoring of metabolites. Glucose biosensors have wide applications in health care institutions or diagnostic labs but have major drawback of enzyme instability over a period of time which has paved way of exploring more biomolecules with different electrochemical properties for efficient glucose sensor development [11]. Electrochemical biosensors are now prepared by amending the surface of carbon/metal electrodes by using enzymes, antibody or DNA [12]. Non-enzymatic biosensors have been created using various synthetic materials, such as ferrocene boronic acid (FcBA) and ferrocene (Fc)-modified boronic acids, which replace proteins. These materials exhibit different stability and selectivity which is attributed to the inclusion of a binding site (boronic acid group) and an electrochemically active element (ferrocene unit). FcBA and its derivatives exhibit a special ability to bind to 1,2- or 1,3-diol groups in sugars and the hydrocarbon chains within the polypeptide structure of glycated hemoglobin (HbA1c) [12]. Recent development in biosensor technology has lead to development of amperometric detection based biosensor with the capability to measure both oxidation/reduction potential providing a diagnostic tool for various clinical abnormalities (detection of uric acid) [13].

## 4. Methods for Synthesizing ZnO Nanostructures

Nanostructures can be synthesized using a variety of techniques, which are generally divided into two main categories: top-down and bottom-up approaches [14]. In the top-down scheme, the procedure begins with a larger bulk material that is gradually reduced or broken down into nanoscale structures using methods such as exfoliation or mechanical grinding. Conversely, the bottom-up method starts at the atomic level, where atoms or molecules aggregate to form nanostructures. The primary methods for developing ZnO nanostructures are outlined below.

### 4.1. Pulsed Laser Deposition

In the pulsed laser deposition (PLD) technique, nanostructures are created by directing a high-energy laser at a bulk crystal. When the laser is focused, the target material is ablated and then deposited as nanostructures on a substrate positioned a short distance away. Kawakami et al. synthesized 99% pure ZnO nanorods using sapphire substrate [15]. These nanorods were synthesized at substrate temperatures ranging from 400 to 700 °C and oxygen pressure between 1 and 10 torr. The rod size can be regulated by varying the duration of deposition, with rods measuring 6 μm in length and 300 nm in diameter obtained after 30 min deposition period. Bae et al. produced nanocones at 580 °C, using pulse energy of 90–100 mJ, in a 10 mTorr O_2_ environment on a substrate [16]. The PLD technique guarantees the stoichiometry and nanostructures with high quality; however, it is constrained by the limited area available for deposition.

### 4.2. Sputtering

Sputtering provides flexibility for growing nanostructures over large substrate areas. In this technique, Zn or ZnO serves as target material, which is showered with high energy Ar ions. In this process, target atoms are knocked out through bombardment and then accumulate on the surface of substrate. The radio frequency (RF) magnetron sputtering method generates a powerful magnetic field around the target, effectively trapping the plasma close to the substrate. Liu and his team utilized RF reactive sputtering, with high-purity zinc (99.99%) as the target and polyvinyl pyrrolidone (PVP) fibers as the substrate, to fabricate nanotubes [17]. The deposition process lasted 20 min at a pressure of 7 × 10^−4^ Pa, followed by annealing at 600 °C. Chiou et al. produced nanowires on Cu/Ti/Si wafers at a pressure of 0.05 torr, achieving diameters of approximately 45 nm after a 5-min deposition and 55 nm after a 30-min deposition [18]. Venkatesh et al. deposited nanorods with a preferred growth orientation in the (002) direction using Ar sputtering at a pressure of 0.01 mbar and 650 °C substrate temperature, with a deposition time of 60 min [19] While sputtering enables large-area deposition, the film quality is generally lower in comparison to that achieved by PLD.

### 4.3. Sol–Gel

In comparison to the chemical vapor deposition method, the sol–gel process is a less costly method suitable for large-area deposition. It supports superior rates of deposition while offering excellent control over the morphology of nanostructures [20]. The sol–gel method generally involves three primary steps. First, the sol is prepared from the initial materials. Next, the sol is deposited onto the substrate using an appropriate method. Finally, a heat treatment is applied to the xerogel film.

Ahn et al. prepared nanorods of ZnO by preparing a sol of a mixture of Zn(NO_3_)_2_·6H_2_O and hexamethylenetetramine (HMTA) in equal parts [21]. The process requires a temperature of around 95 °C, resulting in the production of crystalline nanorods of ZnO having wurtzite structures of hexagonal shape on a SiO_2_/Si substrate. These nanorods’ diameter can be adjusted by adjusting the initial materials’ concentration.

Various precursors like zinc alkoxides, chlorides, nitrates, and zinc acetate dihydrate are used in the sol–gel process. Alkoxides are less favored because they are insoluble in alcohols [22]. Inorganic salts such as nitrates pose challenges during removal of anionic species from the formed product, while employing acetate dihydrate results in by-products having volatile nature. A detailed description of the sol–gel method can be found in the work of Znaidi et al. [23].

### 4.4. Thermal Evaporation

In the thermal evaporation process, the source material is heated to a high temperature, causing it to vaporize and subsequently condense onto the substrate. Interestingly, the substrate doesn’t burn during ZnO nanostructure formation by thermal evaporation because the process is conducted under controlled conditions. The temperature is optimized to vaporize the ZnO precursor while remaining below the substrate’s thermal degradation point. Additionally, the process typically occurs in a vacuum or inert gas atmosphere, preventing oxidative reactions. Substrates are chosen for their thermal stability and are positioned at a distance from the heat source where it remains relatively cooler, sufficient only for ZnO condensation and growth. Umar and colleagues used ZnO powder as the source material to grow ZnO nanorods on a silicon substrate coated with nickel [24]. Nitrogen and oxygen gases were introduced at flow rates of 5–15 sccm and 10–30 sccm, respectively, with deposition times ranging from 30 to 120 min. The ZnO nanorods, measuring 4–5 μm in length and 300–350 nm in diameter, were grown at temperatures between 500 and 550 °C. Bae et al. produced nanowires at temperatures between 800 and 1000 °C, using 500 sccm of argon gas [25]. One advantage of thermal evaporation is that it does not need a catalyst, thereby avoiding unintentional contamination. However, the high temperatures necessary for this process limit the choice of substrates that can be employed to synthesize ZnO nanostructures.

## 5. ZnO Nanostructures for Biosensor Development

Advancements of biosensors makes the use of silicon, gold, silver, copper, quartz or crystal, carbon-based materials such as graphene/carbon nanotubes and glass materials with great sensitivity and specificity. Silicon and gold nanomaterials have greater potential due to their biocompatibility, availability in abundance, and optical, electronic, and mechanical properties [8,26,27]. Biocompatible nanomaterials and biotechnology have made an immense contribution toward the development of enzyme-based biosensors. In the last decade use of ZnO nanostructure in the glucose biosensors development has taken lead. Various morphologies of ZnO nano structures have been adopted as platform for the immobilization of enzyme. ZnO nanocomb which is synthesized by the vapor-phase transport method has been used for the glucose detection [28]. ZnO nanowires are prepared by thermal evaporation technique, wherein powder of ZnS is thermally evaporated under controlled conditions with a catalyst layer of gold thin film [29]. Glucose oxidase peroxidase was immobilized on ZnO nanowires through physical adsorption technique. Various strategies have been used to grow ZnO nano structures on electrodes; a simple method is to build ZnO on the surface of electrode directly. Using this strategy, ZnO nanorods have been fabricated via hydrothermal decomposition directly on the standard gold electrode [30]. Tetragonal ZnO nanostructures which are porous and pyramid-shaped were synthesized through a wet chemical method and utilized in a biosensor incorporating immobilized glucose oxidase.

Electrochemical biosensors, which utilize a variety of components such as nanomaterials, polymers, and microbes, have broad applications across various sectors. Biosensors have emerged as important tools in pathogen detection, molecular diagnostics, food safety, and environmental surveillance [31]. Despite the rapid advancement in biosensor development, clinical uses of biosensors are still rare, with the exception of glucometer. For optimal clinical outcome and public health, rapid diagnosis of infectious diseases is critical. Traditional in-vitro diagnostics consume time and need state-of-the-art laboratories with highly trained staff. Cost-effective devices are in high demand for replacing centralized laboratory testing and providing analytical results directly at the patient’s bedside through point-of-care diagnostics. Due to its distinctive characteristics, nanostructured ZnO offers a stable platform for immobilizing biomolecules while preserving their biological function [32,33]. ZnO nanostructures display useful characteristics that include strong ability of adsorption and high catalytic efficiency. ZnO is recognized as a favourable option for developing biosensors that exhibit improved analytical capabilities [33]. ZnO’s low toxicity, biocompatibility, ease of fabrication, and high electron mobility make it an excellent choice for biosensor development. ZnO nanostructures have been developed in various shapes using different methods namely vapor-liquid-solid, vapor-phase-transport for hydrothermal decomposition and thermal evaporation, respectively [34]. These shapes comprise of nanowires (ZnO NW) [35,36,37,38], nanorods (ZnO NR) [39,40,41,42,43,44,45], combs [46,47], forks [48], fibers [49], flakes [50], waxberries [51], bundles [52], spheres [53,54], composites [55], tetrapods [56,57,58], nanoparticles (ZnO NP) [59,60], tubes (ZnO NT) [61,62], belts [63,64], flowers [65,66,67] and sheets/disks [68,69,70]. Each form of ZnO nanostructure imparts different properties and characteristics to the biosensor. Varying the forms of ZnO nanostructures is essential because different applications require specific physical, chemical, and electronic properties that are influenced by the material’s morphology. Each nanostructure form offers unique advantages:

- **Surface Area**: High surface area structures like nanoparticles or nanotubes enhance interactions with biomolecules, improving sensitivity in biosensors.
- **Electron Transport**: 1D structures like nanorods and nanowires provide superior electron transport, making them ideal for field-effect transistors (FETs) and piezoelectric biosensors.
- **Optical Properties**: Nanodisks with large flat surfaces optimize light absorption and emission, crucial for optoelectronic sensors.
- **Mechanical Stability**: Nanorods and nanowires are mechanically robust, suitable for applications requiring durability.
- **Functionalization**: Different forms allow specific functionalization for targeted applications, such as drug delivery or environmental monitoring.

By tailoring the form, ZnO nanostructures can be optimized for enhanced performance, sensitivity, and efficiency in diverse fields like biosensing, catalysis, and electronics. Table 1 compares the key differences in biosensors based on some important ZnO nanostructure forms.

**Figure 2:**
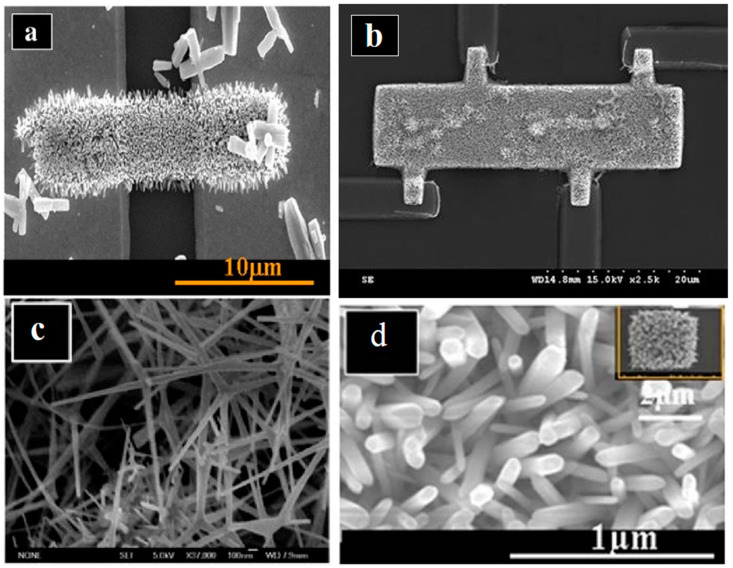
FESEM images of ZnO nanostructures with various shapes: (**a**) low magnification image of ZnO nanorods bridge grown on and between two-point probe electrodes, (**b**) low magnification image of ZnO nanorod arrays grown directly on a fourpoint probe, (**c**) high magnification image of ZnO nanotetrapods, and (**d**) high magnification images of ZnO nanorod arrays (NRAs) grown on square patterns. (**a**–**d**) have been adapted from [39,40,41,55].

Qurashi et al. [71] directly assessed the electrical properties of as-grown nanorods (NRs), avoiding the conventional pick-and-place method typically used in nanodevice fabrication. This was accomplished by directly growing ZnO nanorod arrays on an on-chip system, where site-selective growth formed nanorod-array bridges between two-point gold electrodes, as illustrated in Figure 2a. In a separate study, Qurashi et al. [40] developed a method to measure the electrical properties of aligned ZnO nanorod arrays (NRAs) grown directly on a pre-patterned four-point probe system in solution. Figure 2b displays low-magnification field emission scanning electron microscopy (FESEM) images of ZnO nanorod arrays grown on the four-probe electrode system, with each strip pattern accurately aligned between the four electrodes. Furthermore, highly crystalline, high aspect-ratio ZnO nanotetrapods were synthesized rapidly using an innovative microwave technique [55], as seen in Figure 2c. These ZnO nanotetrapods were utilized for detecting bisphenol A (BPA), with the ZnO nanotetrapod-modified electrode sensor significantly increasing the anodic current of BPA while reducing the detection limit.

Qurashi et al. [39] developed a novel seedless hybrid technique for the selective growth of ZnO nanorod arrays on specific regions of thin cover glass substrates. Pattern transfer onto the seedless substrate was achieved using electron-beam lithography, followed by a solution-based method for the bottom-up growth of ZnO nanorod arrays on the patterned areas. Figure 2d presents an FESEM image of ZnO nanorod arrays selectively grown on cover glass substrates, with each nanorod exhibiting a hexagonal nanotip. The inset in Figure 2d shows a square pattern measuring 4 μm × 4 μm.

**Figure 3:**
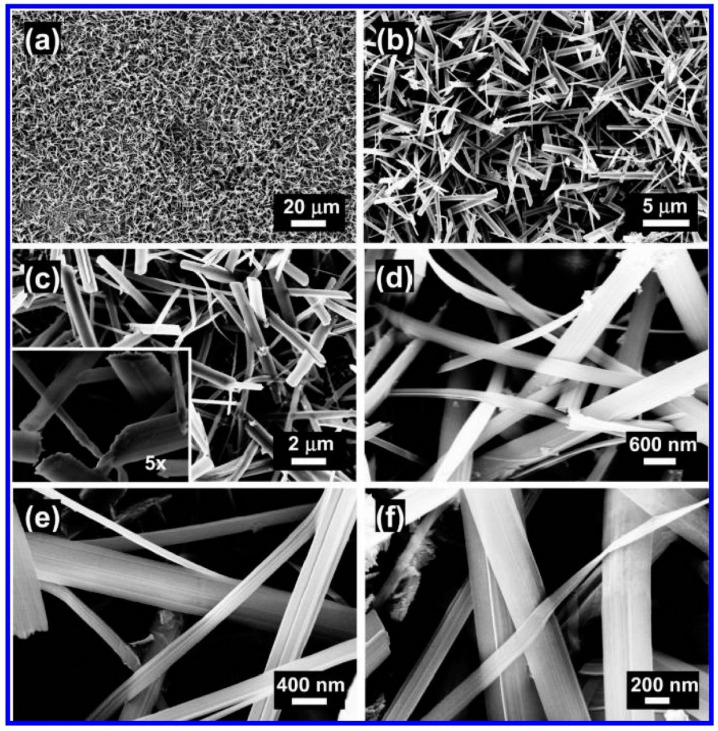
(**a**–**f**) SEM images of ZnO nanobelt-like structures being electrodeposited on a polyethylene terephthalate (PET) conducting substrate at 0 °C in 0.1 M $Zn{{(NO}_{3})}_{2}\cdot 6H_{2}O$ mixed with 0.1 M KCl electrolyte solution for a duration of 180 min. Adapted from [63].

Pradhan et al. synthesized single-crystal ZnO nanobelt-like structures (NBS) with dimensions ranging from 5–10 μm in length, 50–400 nm in width, and 20–100 nm in thickness using a single-step electrochemical deposition method at 0 °C [63]. The NBS were reported to grow uniformly across the entire substrate, as depicted in the SEM images (see Figure 3a). These structures appear slender and flexible, as shown in Figure 3b–f, and feature an oval-shaped cross-section, with the central region being thicker than the edges (refer to the inset of Figure 3c).

## 6. Application of ZnO Nanostructures in Various Biosensors

ZnO nanostructures are highly suitable for detecting a range of analytes such as glucose, phenol, and other biomolecules. Here, we explore the use of ZnO nanostructures in various types of biosensors.

### 6.1. Glucose Biosensors

Glucose biosensors employing glucose oxidase (GOx) as the enzyme have found extensive applications in clinical diagnostics, such as diabetes detection, and in the food industry. Early ZnO-based biosensors utilized physically adsorbed glucose oxidase on single-crystal ZnO nanocombs [28] and nanowires [29], exhibiting high sensitivity due to the strong binding affinity between glucose oxidase and glucose. Other biosensors were developed by electrostatically immobilizing glucose oxidase onto ZnO nanorod matrices [30], achieving remarkable stability, high sensitivity, and rapid response times for glucose detection, facilitated by direct electron transfer between the electrode’s active sites and the immobilized enzyme. Additionally, a variety of glucose biosensors leveraging glucose oxidase have been engineered, including those utilizing tetragonal pyramid-shaped porous ZnO nanostructures on glassy carbon electrodes, ZnO nanorods integrated with Au nanocrystals, Co-doped ZnO nanoclusters, carbon-decorated ZnO nanowire arrays, and vertically aligned ZnO nanowires coated with a ZnS nanocrystal monolayer. ZnO-based glucose biosensors incorporating multiwalled carbon nanotubes have also shown a rapid glucose response, with a detection limit as low as 2.22 μM [72].

**Figure 4:**
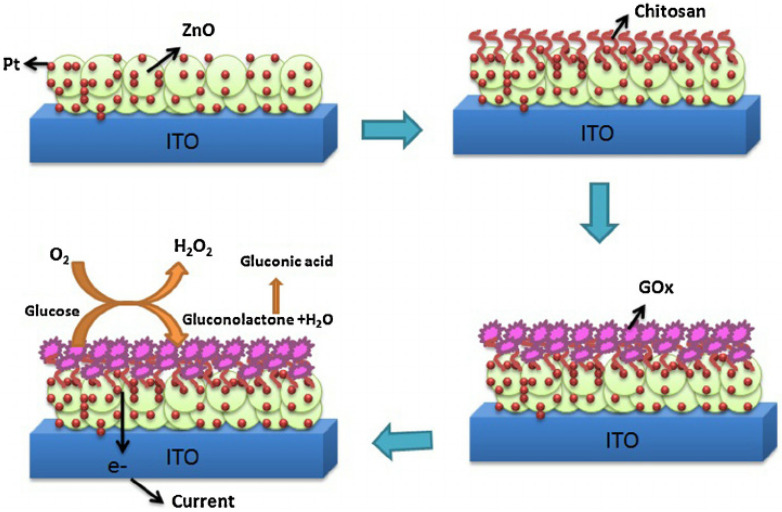
Sketch displaying ZnO/Pt/CS biosensor fabrication. Adapted from [73].

The use of nanoparticles (NPs) in fabricating biosensors is an emerging area of research. Recently, Anusha et al. developed an enzymatic glucose biosensor by dispersing Pt nanoparticles over a ZnO nanoporous structure on an FTO substrate using commercially available Pt paste [73]. A chitosan (CS) layer was then coated onto the ZnO/Pt surface to create a heterostructured electrode. Glucose oxidase (GOx) was immobilized by applying a suitable amount onto the ZnO/Pt/CS electrode, drying it at room temperature, and storing it in the dark at 4 °C. The fabrication process of the ZnO/Pt/CS electrode is schematically illustrated in Figure 4. The ZnO/Pt/CS heterostructure conjugated with GOx demonstrated superior analytical performance, offering higher sensitivity ($62.14\;\mu A\;{mM}^{-1}\;{cm}^{-2}$) over a broad linear range and a lower detection limit compared to the bare ZnO/CS/GOx biosensor.

**Figure 5:**
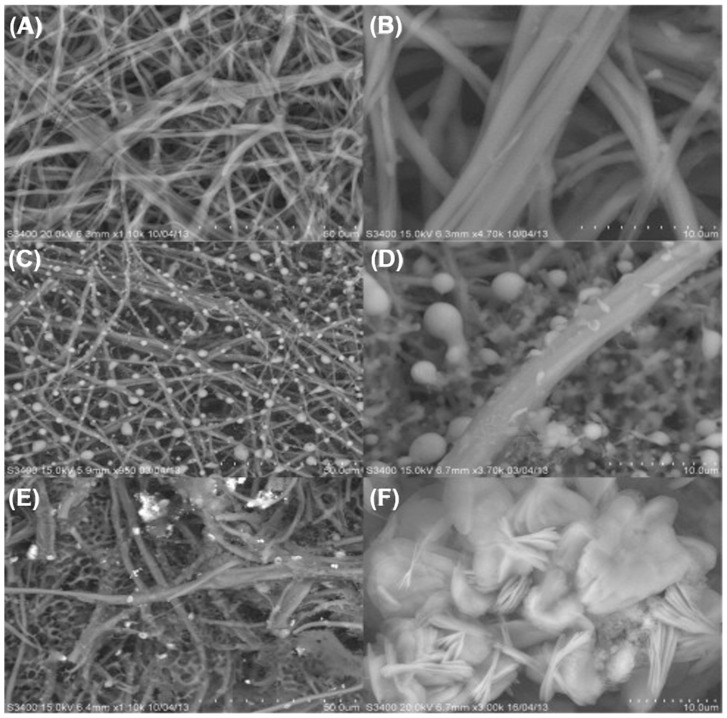
SEM images of (**A**,**B**) egg shell membrane [ESM], (**C**,**D**) egg shell membrane immobilized with glucose oxidase [GOx/ESM], and (**E**,**F**) egg shell membrane immobilized with ZnO nanoparticles and GOx [GOx/ZnONPs-[EMIM][Otf]/ESM]. Adapted from [74].

Additionally, Aini et al. fabricated an electrochemical glucose biosensor by combining ZnO nanoparticle films with glucose oxidase (GOx) immobilized on an eggshell membrane (ESM) [74]. The fabrication involved depositing an ionic liquid (IL), such as 1-ethyl-3-methylimidazolium trifluoromethanesulfonate ([EMIM][Otf]), ZnO nanoparticles (ZnO NPs), and an ESM onto a modified glassy carbon electrode (GCE). GOx was covalently immobilized onto the ESM using glutaraldehyde as a cross-linking agent. Figure 5 shows SEM images of the ESM membranes. Figure 5A,B present the morphology of ESM under SEM, while Figure 5C,D display well-dispersed GOx on ESM without nanoparticles. Figure 5E,F reveal dense particle-like clusters formed after the addition of ZnO NPs.

### 6.2. Phenol Biosensors

Phenol biosensors have been created using the electrostatic interactions involving positively charged ZnO nanostructures and active tyrosinase enzymes [33]. They are fabricated by coating Nafion on ZnO nanorods and immobilizing tyrosinase via electrostatic interactions [75]. Tyrosinase was attached to the ZnO nanorods, exhibiting excellent biological activity with a rapid response time of 5 s. The biosensor showed a linear detection range from 0.02 to 0.18 mM, and a low K_M_ value of 0.24 mM indicated high affinity between tyrosinase and phenol on the ZnO nanorods [75]. A new biosensor utilizing ZnO nanorod microarrays on boron-doped nanocrystalline diamond substrates has been recently described [76]. Due to covalent immobilization of tyrosinase onto ZnO nanorods, this sensor demonstrated exceptional sensitivity to phenol. Another biosensor design involved encapsulating tyrosinase within a CNT-ZnO-nafion composite film on GCE, showcasing remarkable sensitivity and an ultra-fast response time of 2 s [77].

**Figure 6:**
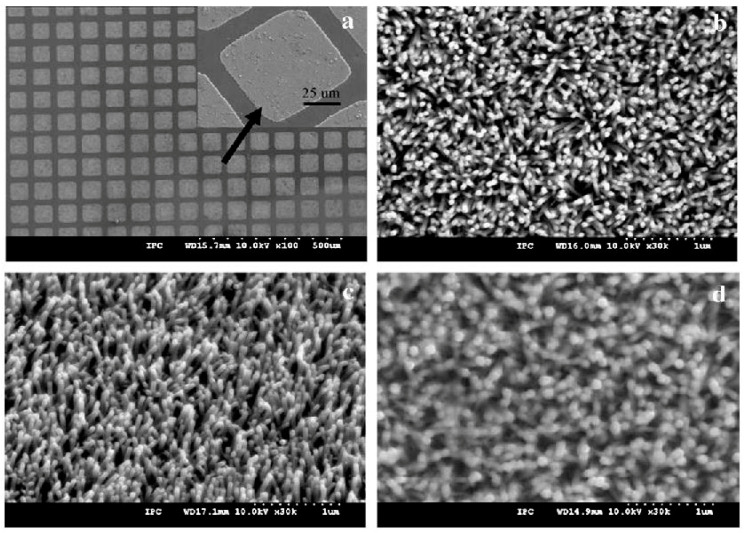
SEM images of (**a**) ZnO nanorod microarrays deposited on BDND thin film substrate (the inset displays the SEM image of square-shaped ZnO nanorod array), (**b**) ZnO nanorod arrays top view, (**c**) its tilt view, and (**d**) functional ZnO nanorod array. Adapted from [76].

Zhao et al. [76] introduced a tyrosinase biosensor in which tyrosinase was covalently bound to biofunctional ZnO nanorod microarrays grown on a boron-doped nanocrystalline diamond (BDND) thin film electrode. Figure 6a shows the ZnO nanorod microarrays on the BDND thin film surface, where the edges of the micropattern lines appear almost straight and uniform. Figure 6b,c present the top and tilted views of the ZnO nanorod arrays grown on the BDND substrates, with the ZnO nanorods having a diameter of approximately 50 nm. The SEM image of the ZnO nanorods became blurry after modification with silica through a co-condensation process, as seen in Figure 6d, due to the non-conductive silica coating.

### 6.3. Cholesterol Biosensors

ZnO nanostructures have been employed as a foundation for creating cholesterol biosensors, utilizing cholesterol oxidase. This enzyme is crucial for the rapid and convenient detection of cholesterol, a primary component of nerve and brain cells in humans [33]. The rapid and accurate determination of cholesterol is crucial, as elevated serum cholesterol levels are associated with several clinical conditions, including coronary artery disease, arteriosclerosis, hypertension, and cerebral thrombosis.

Cholesterol oxidase (ChOx) is the most widely used enzyme in the fabrication of cholesterol biosensors. It is a flavin enzyme (flavin-adenine-dinucleotide) that produces hydrogen peroxide through the following reaction.

(1) $$Cholesterol+O_{2}\to 4-Cholesten-3-one+H_{2}O_{2}$$

(2) $$H_{2}O_{2}\to O_{2}+2H^{+}+2e^{-}$$

Cholesterol is catalyzed by ChOx in the presence of oxygen, leading to the production of hydrogen peroxide. The electrooxidation current of hydrogen peroxide is then measured by applying an appropriate potential to the system, which forms the basis for cholesterol detection. Cholesterol oxidase holds significant industrial and commercial value due to its application in bioconversions for determining free or total serum cholesterol levels in serum samples. Singh et al. demonstrated the successful use of a nanoporous ZnO film, fabricated via the rf sputtering technique on a gold electrode, for cholesterol detection [78]. The biosensor showed a strong linear response to cholesterol concentrations ranging from 25 to 400 mg/dL.

**Figure 7:**
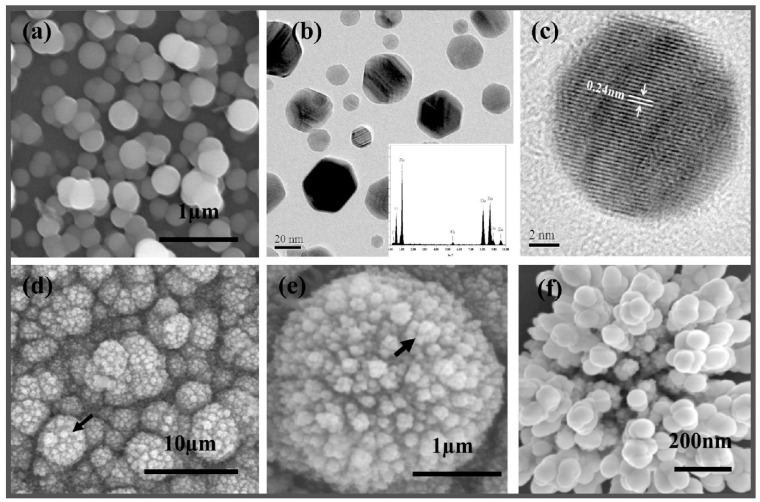
(**a**) ZnO nanospheres SEM image, (**b**) TEM image with ZnO nanospheres EDS. (**c**) HRTEM image of individual ZnO nanosphere. (**d**–**f**) SEM images of the as-deposited Pt-incorporated ZnO nanospheres. (**d**) Low-magnification image of spherical agglomerates of nanospheres. (**e**) Individual agglomerate sphere magnified from the arrow point in (**d**). (**f**) Pt-ZnO nanospheres on the surface of self-assembled microspheres magnified from the arrow point in (**e**). Adapted from [79].

Later on, Ahmed et al. revealed amperometric biosensor with high sensivity utilizing hybrid nanospheres composed of Pt-incorporated fullerene-like ZnO. [79]. They fabricated Pt-ZnO nanospheres (PtZnONS) with diameters ranging from 50 to 200 nm using an electrodeposition method on a glassy carbon electrode (GCE). The biosensor demonstrated excellent and consistent sensitivity of 1886.4 $\mu A\;{cm}^{-2}\;{mM}^{-1}$ to cholesterol having less than 5 s response time and a linear range of 0.5 to 15 µM. Figure 7 shows the characterization of Pt-ZnO nanospheres. Figure 7a shows ZnO nanosphere morphology, having a mean diameter ranging between 10 and 200 nm. Figure 7b shows the transmission electron microscopy (TEM) image of the ZnO nanospheres, along with the energy dispersive X-ray spectroscopy (EDS) inset. The high-resolution transmission electron microscopy (HRTEM) image in Figure 7c reveals the single-crystalline structure of the ZnO nanospheres, with an interplanar distance of 0.24 nm, corresponding to the (101) planes in wurtzite ZnO. Figure 7d illustrates the overall morphology of the Pt-incorporated ZnO nanospheres after deposition, consisting of densely packed spherical agglomerates of PtZONS, with average diameters ranging from 0.2 to 3.5 µm. A closer SEM image of a single agglomerate, highlighted by the arrow in Figure 7e, reveals that the PtZnONS formed multiple smaller spherical agglomerates on the surface of the larger agglomerate. Upon further magnification of the arrow in Figure 7e, a fullerene-like spherical structure of PtZnONS is observed, with diameters between 50 and 200 nm, as shown in Figure 7f.

### 6.4. Urea Biosensors

**Figure 8:**
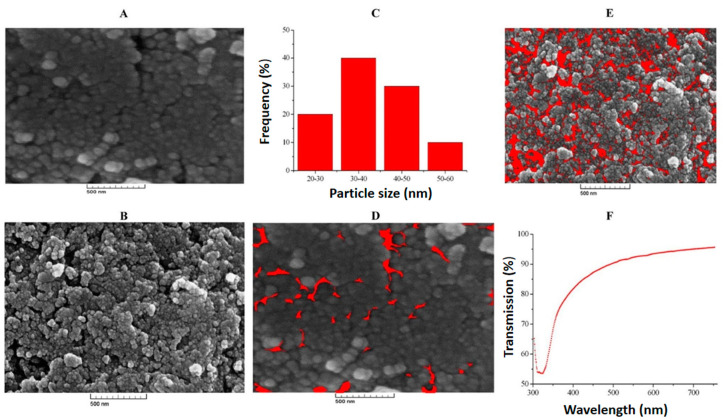
FESEM images (**A**) pre- and (**B**) post-annealing, (**C**) histogram of particle size distribution, pore size distribution (**D**) pre- and (**E**) post-annealing, and (**F**) UV transmission spectrum of ZnO nanoporous thin film. Adapted from [84].

ZnO-based biosensors have been developed for detecting compounds such as uric acid and urea, using the enzyme uricase [80,81]. The first urea biosensor utilizing ZnO nanomaterials was reported in 2009. In this study, urease was immobilized onto ZnO, both with and without chitosan, on indium-tin-oxide-coated glass through physical adsorption [82,83]. Another biosensor for amperometric urea detection was created by electrostatically attaching urease to ZnO nanorods grown on indium-tin-oxide-coated glass, achieving a response time of 3 s and a detection limit of 0.13 mM urea [84]. More recently, ZnO nanowire arrays grown on gold-coated plastic substrates have been used in a urea biosensor, with urease immobilized via physical adsorption [84]. Rahmanian et al. demonstrated fabrication of a urea biosensor based on semiconductor technology [84]. Field emission-scanning electron microscopy (FE-SEM) images of the ZnO films pre- and post-annealing are displayed in Figure 8. In the pre-annealing process, the layer seems to be flat and comprised of long grains forming a dense film (refer Figure 8A). Figure 8B shows the surface morphology of the ZnO film via FE-SEM technique after annealing. The FE-SEM images revealed that the grain size of the ZnO nanostructured film ranged from 20 to 60 nm, with an average size of approximately 40 nm. The particle size distribution histogram of the ZnO nanostructured film is shown in Figure 8C. Figure 8D,E present the surface analysis of the FE-SEM images of the ZnO film before and after annealing, performed using Image Analysis Software from Nahamin Pardazan Asia Co. The surface analysis indicated that the surface porosity increased from 0.09 to 0.45 μm^2^ after annealing, which is believed to play a key role in enzyme immobilization. The optical transmission spectrum of the ZnO nanoporous film is shown in Figure 8F.

### 6.5. H_2_O_2_ Biosensors

**Figure 9:**
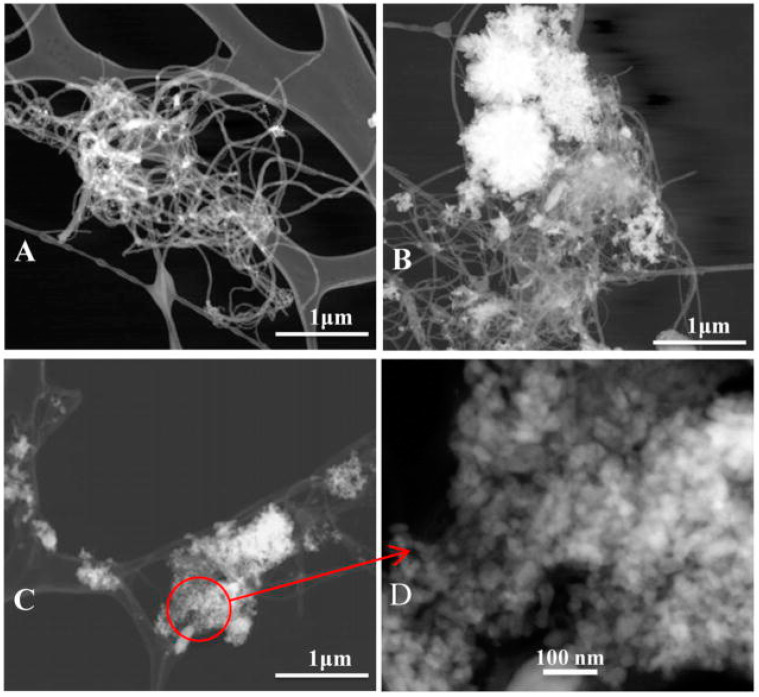
STEM images of (**A**) ZnO/COOH-MWNTs at 40 °C, (**B**) ZnO/COOH-MWNTs at 60 °C, and (**C**,**D**) ZnO/COOH-MWNTs at 90 °C. Adapted from [90].

Presently, detecting H_2_O_2_ is essential due to its significant applications in the food industry, clinical diagnostics, and environmental monitoring [85]. Horse radish peroxidase (HRP) enzyme is typically employed for sensing $H_{2}O_{2}$ owing to its high selectivity [86]. Liu et al. reported a biosensor fabricated with nanosized flower-like ZnO to detect $H_{2}O_{2}$ with high stability and reproducibility [87]. ZnO was incorporated into a chitosan solution to form a ZnO/chitosan composite matrix, which was then immobilized with horseradish peroxidase (HRP). Using hydroquinone as a mediator, the biosensor exhibited a rapid response time of less than 5 s, a linear range of $1.0\times 10^{-5}-1.8\times 10^{-3}$ M H_2_O_2_, and a detection limit of 2.0 μM, with a signal-to-noise ratio of 3. Later, Cao et al. developed an H_2_O_2_ biosensor by co-immobilizing HRP with waxberry-like ZnO microstructures, composed of 8–10 nm nanorods, onto the surface of a glassy carbon electrode (GCE) [88]. Another biosensor based on ZnO nanorods, grown on a gold wire, was created by alternately immobilizing poly(sodium 4-styrenesulfonate) (PSS) and HRP on the ZnO nanorods, as documented by Gu et al. The multilayered HRP sensors showed a wide linear range, a low detection limit, and a significant reduction in response time to approximately 5 s [89]. More recently, Wayu et al. synthesized zinc oxide (ZnO) nanoparticles and demonstrated their attachment to multiwalled carbon nanotubes (MWCNTs), resulting in a composite with a unique synergistic response [90]. They controlled the morphology and size of the ZnO nanostructures using the hydrothermal synthesis technique and by varying the hydrothermal treatment temperature before attaching them to carboxylic acid-functionalized MWCNTs for H_2_O_2_ sensing applications. The study revealed that the electrocatalytic activity was strongly influenced by the shape of the nanosized ZnO, and the activity varied with pH, peaking at pH 7.4. A stable, linear response for H_2_O_2_ concentrations was reported in the range of 1–20 mM. The scanning transmission electron microscopy (STEM) images of the ZnO/COOH-MWNTs composite at different hydrothermal treatment temperatures of ZnO nanostructures are shown in Figure 9. The COOH-MWNTs nanocomposite created from ZnO synthesized at 40 °C shows ZnO sparsely distributed on the MWNT surface (Figure 9A). At 60 °C, more of the MWNT surface is covered, though approximately half of it remains exposed (Figure 9B). When the hydrothermal treatment temperature is increased to 90 °C, the MWNT surface is fully covered, as shown in Figure 9C,D.

### 6.6. Electrochemical Immunosensor

**Figure 10:**
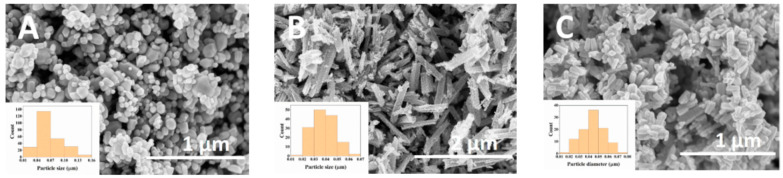
SEM images of (**A**) No. 1, (**B**) No. 2, and (**C**) No. 3 ZnO samples. Adapted from [93].

**Figure 11:**
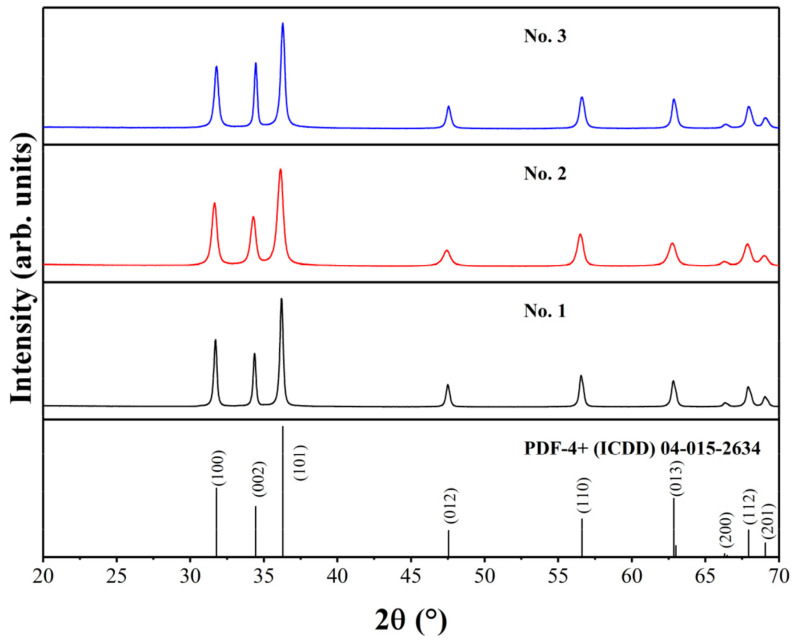
XRD of various ZnO samples. Adapted from [93].

Despite numerous studies on biosensors utilizing one-dimensional ZnO nanostructures for detecting enzymes linked to various diseases, there are relatively few publications focusing on ZnO-based detection of cells and cancer biomarkers [91]. As a result, developing point-of-care immunosensing devices for early cancer detection could pave the way for innovative treatment strategies and improve patient care through real-time monitoring. However, achieving these goals requires meeting several key criteria: rapid, label-free, and selective detection of cancer markers; cost-effectiveness; compact sensor design; and portability. Thus, to adhere to these requirements, Drobysh et al. utilized carbon electrodes fabricated via screen printing, ensuring repeatability, cost-effectiveness, and disposability [92] Their research examined the structural, morphological, and photoluminescent properties of ZnO nanostructures and subsequently led to the creation of a novel electrochemical immunosensor [93]. This sensor utilized screen printing carbon electrodes (SPCEs) that were enhanced with specific ZnO nanostructures for detecting anti-prostate-specific antigen (anti-PSA) in PSA samples, as displayed in Figure 10. The minimum detectable concentration and minimum quantifiable concentration of anti-PSA were established as 1.35 nM and 4.08 nM, respectively, for compact spherical ZnO nanostructures, and 2.36 nM and 7.15 nM, respectively, for ZnO nanostructures having rod-shape. Their XRD analysis closely matches the standard hexagonal ZnO pattern, as shown in Figure 11. The No. 2 sample exhibits the broadest peaks, indicating 18 nm crystallite size. Comparatively, the No. 3 sample shows larger crystallites, approximately 24 nm in size, while the No. 1 sample displays even larger crystallites, measuring around 30 nm.

## 7. Conclusions, Challenges and Future Prospects

Nanostructured materials have revolutionized the field of biomedicine and witnessed overwhelming progress during the past few decades. Due to the advancement of novel synthesis methodologies and better characterization techniques that resulted in the invention of newer functionalities, nanostructured materials have gained an extensive research focus. Nanomaterials exhibit unique properties that differ from those of both molecules and bulk solids due to their diminutive sizes, often several orders of magnitude smaller than human cells. This enables them to interact remarkably well with biomolecules, both on the surface and within cells. Among all nanomaterials, ZnO nanostructures have established themselves as a potential biomaterial for biosensor fabrication. Due to the enthralling physicochemical properties and great potential for various biomedical applications, ZnO nanostructures emerged to be a prominent biocompatible and biodegradable candidate for biosensing applications. ZnO nanostructures have supremacy over nanostructures of other materials since they have high isoelectric point, better charge transfer properties, facile synthesizing processes and large surface to volume ratio. These nanostructures exhibit faster responses and enhanced sensitivity in biosensor applications as compared to planar configurations due to their exceptional electrical, optical and physico-chemical properties. Now, low-cost biosensors are commercially realizable because of the ease of fabrication of innumerous ZnO based nanostructured matrices by inexpensive processes. Moreover, the physicochemical properties of these nanostructures can be significantly reinforced by doping ZnO with noble metals, which is pivotal for their practical feasibility.

This review has attempted to survey most of the reported ZnO nanostructure-based biosensors in light of the film synthesis and its morphological perspective. The focus has been on ZnO nanostructure fabrication methods, performance of the biosensors, and enzyme immobilization techniques. It is evident that the fabrication techniques of these nanostructured thin film are already present in the laboratories, however, the most crucial present-day challenge is the large-scale production of these nanostructures with well-controlled dimensions and morphologies. Furthermore, rigorous research is entailed for fabricating the ZnO nanostructures with reproducibility. In addition, the issue of interaction of 1D ZnO nanostructures with a variety of biomolecules needs to be probed extensively, as its development as biosensors is still in the embryonic stage. Additionally, effectively integrating the fabricated ZnO nanostructures with commercial detection systems for practical biosensor applications remains a significant challenge. In the time ahead, ZnO nanostructure–based biosensors are expected to be propitious for diagnosing and monitoring infectious disease, point-of-care diagnostics, controlling the pharmokinetics of drugs, detecting cancer, and disease biomarkers, detection of pathogenic organisms, food safety, examining breath, urine and blood for drugs of abuse. Despite their advantages, ZnO nanostructures face several challenges in biosensor applications. One major issue is reproducibility; achieving consistent and reproducible synthesis of ZnO nanostructures can be difficult, which in turn impacts the sensor’s performance. Another significant challenge is surface functionalization; effectively functionalizing ZnO nanostructures to immobilize bioreceptors is crucial for ensuring optimal biosensor performance. Additionally, the stability of ZnO nanostructures in biological environments is a concern, as they can degrade or dissolve in certain biological conditions, which affects their long-term stability and reliability. Future research is needed to improve the reusability of these ZnO nanostructured biosensors by developing simple immobilization techniques and upgrading the components’s stability. The future also offers the potential for direct electron transfer between enzymes and ZnO nanostructure-based electrode surfaces, eliminating the need for co-substrates or mediators. This advancement could pave the way for the development of reagentless biosensors that are non-toxic, biosafe, and suitable for implantation in the human body.

## Figures and Tables

**Table 1:** Comparison of biosensors based on ZnO nanostructure forms.

ZnO Nanostructure	Form	Surface Area	Advantages	Applications	Limitations
Nanoparticles	Spherical/Quasi	High	High surface reactivity, easy functionalization	Enzyme-based biosensors, immunosensors	Lower mechanical stability, agglomeration
Nanorods	1D (High aspect ratio)	Moderate	High alignment, mechanical stability	Piezoelectric biosensors	Requires alignment, challenging functionalization
Nanowires	1D (Uniform)	High	Superior electron transport, flexibility	FET-based biosensors	Complex fabrication, bending issues
Nanodisks/Nanosheets	2D (Flat)	High	Large functionalization area, photocatalytic properties	Optoelectronic biosensors	Limited mechanical robustness
Nanotubes	Hollow tubular	High (hollow core)	Good for immobilization, drug delivery	Ion/bio molecule sensors	Complex synthesis, structural integrity issues

## Data Availability

The data presented in this study are available from the corresponding author upon reasonable request.
